# Treatment of Tularemia in Pregnant Woman, France

**DOI:** 10.3201/eid1906.130138

**Published:** 2013-06

**Authors:** Charlotte Dentan, Patricia Pavese, Isabelle Pelloux, Sandrine Boisset, Jean-Paul Brion, Jean-Paul Stahl, Max Maurin

**Affiliations:** Centre Hospitalier Universitaire de Grenoble, Grenoble, France (C. Dentan, P. Pavese, I. Pelloux, S. Boisset, J.-P. Brion, J.-P. Stahl, M. Maurin);; Université Joseph Fourier–Grenoble 1, Grenoble (S. Boisset, J.-P. Stahl, M. Maurin)

**Keywords:** tularemia, Francisella tularensis, pregnancy, macrolides, France, antimicrobial resistance, bacteria, zoonoses, azithromycin

## Abstract

A pregnant woman who had oropharyngeal tularemia underwent treatment with azithromycin and lymph node resection and recovered without obstetrical complication or infection in the child. Azithromycin represents a first-line treatment option for tularemia during pregnancy in regions where the infecting strains of *Francisella tularensis* have no natural resistance to macrolides.

Tularemia is a bacterial zoonotic disease caused by a gram-negative coccobacillus, *Francisella tularensis*; the most virulent subspecies, *tularensis* (type A), is found in North America, but subspecies *holarctica* (type B) occurs throughout the Northern Hemisphere (*1*–*4*). In France, human cases are most often sporadic and occur predominantly in the eastern, southeastern, and western parts of the country (*1*). 

*F. tularensis* has several animal reservoirs, including rodents and lagomorphs (hares and rabbits). Domestic animals may be infected with *F. tularensis* through contact with the wildlife fauna and, occasionally, transmit the disease to humans. Human contamination occurs following direct contact with infected animals, through contaminated environments or arthropod bites (*2*).We describe the diagnosis and treatment of tularemia in a pregnant woman in France.

## The Case

In late February 2006, a 27-year-old pregnant woman, at 6 weeks’ gestation, was referred to Grenoble University Hospital, Grenoble, France, for investigation of persistent left cervical lymphadenopathy with fever. She had no history of severe infection or underlying illness. The patient lived in a farm in the French Alps. The lymphadenopathy occurred 3 weeks earlier, along with a sore throat, and persisted despite 10 days’ treatment with amoxicillin (3 g daily). 

At admission, the patient was febrile (38°C) and had a tender, swollen, submaxillary cervical lymph node on the left side. Examination of the oral cavity showed no abnormalities. Magnetic resonance imaging of the left cervical region showed a large mass extending from the parotid region to the submandibular region, with hypo- and hypersignals in T1- and T2-weighted imaging, respectively. 

Lymph node tissue was obtained by needle aspiration, and examination revealed nonspecific lesions of lymphadenitis. Laboratory test results showed moderate inflammatory syndrome (C-reactive protein 67 mg/L). Serologic results were negative for HIV, hepatitis B and C viruses, rubella virus, *Treponema pallidum* (syphilis), *Coxiella burnetii* (Q fever), and *Borrelia*, *Bartonella*, *Brucella*, and *Legionella* spp. and showed only residual IgG-type antibodies against cytomegalovirus, Epstein-Barr virus, herpes simplex virus, parvovirus, *Mycoplasma pneumoniae*, and *Chlamydophila pneumoniae*. 

Because we suspected severe *Streptococcus pyogenes* infection, a second course of amoxicillin was administered for 13 additional days. After initial clinical improvement, the patient relapsed, and the lymphadenopathy evolved to suppuration and necrosis. In March 2006, a large amount of pus was surgically drained from the site, and inflamed lymph nodes were removed. These unusual clinical features led us to suspect tularemia. 

The patient denied receiving any tick bite but reported that she regularly fed domestic rabbits in cages. Her symptoms began a few days after she killed and skinned rabbits, which were then kept frozen but not eaten by the family. 

Examination of the removed lymph node samples confirmed the presence of nonspecific lymphadenitis. Routine cultures and mycobacterial cultures remained sterile, but *F. tularensis* DNA was detected in lymph node samples by using specific real-time PCR targeting the gene encoding a 23-kDa surface protein (*5*). PCR amplification and sequencing of the 16S rDNA–23S rDNA intergenic spacer region (*5*) directly from lymph node tissue confirmed the presence of DNA from *F. tularensis* subsp. *holarctica*. Serum samples collected in late February and on March 15, 2006, were tested by using an immunofluorescence assay and a homemade *F. tularensis* antigen (*5*). Both samples were positive, with IgM and IgG titers of 320 (cutoff titers of >160). 

Because the patient was pregnant and tularemia in France has been caused only by biovar 1 strains of *F. tularensis* subsp. *holarctica* (*2*), which are naturally susceptible to macrolides, she was treated with azithromycin (500 mg/d for 6 weeks). She recovered, with no complications for herself or her baby. 

The patient reported that the rabbits were reared outdoors, in floor-level, wire-mesh cages; therefore, we suspected the animals were infected with *F. tularensis* through contact with wild fauna. Attempts to detect *F. tularensis* in pieces of the frozen rabbits by culture and PCR tests were unsuccessful. All family members of the patient also tested negative for *F. tularensis*.

## Conclusions

Clinical symptoms of tularemia are primarily related to the portal of entry of bacteria, the *F. tularensis* strain virulence, and the immune status of the patient. The incubation period is usually 1–3 days but may last up to 15 days (*3*,*6*). The primary clinical forms are glandular and ulceroglandular (skin inoculation), oculoglandular (conjunctival inoculation), oropharyngeal (oral contamination), pneumonic (inhalation of an infected aerosol), and typhoidal (various modes of infection) (*2*,*7*). Tularemia may be severe and even fatal; patients with lymphadenopathy may experience lymph node suppuration in 30% of cases (*1*). There have been no reports of human-to-human transmission. 

Our patient’s symptoms were fever, pharyngitis, and cervical lymphadenopathy; treatment with a β-lactam drug did not improve her condition, which rapidly evolved to local suppuration, a sign that should prompt physicians to consider oropharyngeal tularemia in disease-endemic regions. Because tularemia cases remain extremely rare in the French Alps, where the patient lived, this diagnosis was not considered at the time of the first medical consultation. 

Because the rabbit meat was not consumed by the patient, we suspected she became infected at the time she skinned these animals. *F. tularensis* resists low temperatures, including freezing (*2*,*6*). However, the animals had no overt disease at the time they were killed, so low bacterial inoculum may explain why the tests performed on rabbit meat had negative results.

Diagnosis of tularemia is often made by serologic tests (*3*), although these are negative during the first 2 weeks following the onset of symptoms (*1*). In the case described here, the same high antibody titers were obtained in 2 serum samples taken 2 weeks apart, which indicates that the peak secretion of specific antibodies was achieved. Culture of *F. tularensis* from clinical samples remains poorly sensitive, but PCR-based testing of pharyngeal swab specimens or lymph node suppurations or biopsy specimens enables rapid diagnosis of oropharyngeal tularemia and identification of the *F. tularensis* subspecies involved (*1*,*4*,*5*,*7*,*8*).

A few tularemia cases occurring in pregnant women have been reported (*9*). Severe illness or death caused by infection with *F. tularensis* could be a risk for a pregnant woman or her fetus; the role of *F. tularensis* as an agent of abortion and intrauterine death is well recognized in sheep (*10*) but not in pregnant women. A major difficulty in this instance was the choice of the antimicrobial drug regimen, because first-line antibiotics currently recommended for treatment of tularemia, including the aminoglycoside gentamicin, fluoroquinolones, and tetracyclines (*1*,*2*,*7*,*8*), may be toxic for pregnant women or fetuses. No treatment recommendation for tularemia during pregnancy is available (*9*). 

This patient recovered after removal of suppurated lymph nodes and treatment with azithromycin. The pregnancy outcome was favorable, and the patient and the infant were healthy at 12-month follow up. Macrolides are usually not recommended for treatment of tularemia patients (*2*,*7*,*8*), especially because *F. tularensis* subsp. *holarctica* biovar 2 strains, mainly found in Eastern Europe and Asia, are naturally resistant to macrolides (*11*–*14*). The ketolides (e.g., telithromycin) are highly active against *F. tularensis* in vitro (*11*,*15*), but their use in pregnant women is currently discouraged. This case emphasizes the usefulness of azithromycin as a first-line treatment for tularemia in pregnant women in areas where infections caused by biovar 2 strains of *F. tularensis* subsp. *holarctica* do not occur.
